# Does Treatment Impact Health Outcomes for Patients After Acute Coronary Syndrome?

**DOI:** 10.3390/ijerph120606136

**Published:** 2015-05-29

**Authors:** Jelena Umbrasienė, Giedrius Vanagas, Jon Venclovienė

**Affiliations:** 1 Department of Preventive Medicine, Lithuanian University of Health Sciences, 57-302 Šiaurės ave, Kaunas LT-49264, Lithuania; E-Mail: giedrius.vanagas@lsmuni.lt; 2 Institute of Cardiology, Lithuanian University of Health Sciences, 17 Sukilėlių ave, Kaunas LT-50009, Lithuania; E-Mail: j.vencloviene@gmf.vdu.lt

**Keywords:** cardiovascular mortality, evidence-based treatment

## Abstract

*Background*: Mortality rates for acute coronary syndrome (ACS) patients are still very high all over the world. Our study aimed to investigate the impact of ACS treatment on cardiovascular (CV) mortality eight years following ACS. *Methods*: A retrospective cohort study with a total of 613 patients was used. The data was collected from databases and medical records. An evidence-based treatment (EBT) algorithm was used based on the ESC guidelines. Logistic regression analysis and standardized odds ratios with 95% confidence interval (CI) were used for the risk assessment, with a *p* level < 0.05 considered as significant. *Results*: The median follow-up time in this study was 7.6 years. During follow-up 48.9% of the patients (*n* = 300) died from CV and 207 (69%) for a relevant reason. For monotherapy ACE inhibitors and β-blockers, and for fixed dose combined drugs ACE inhibitors and diuretics, were most frequently used. EBT was provided to 37.8% of patients. The EBT use (HR 0.541, CI 0.394–0.742, *p* < 0.001) during follow-up period was important for reducing CV mortality in ACS patients. *Conclusions*: The combined use of EBT significantly improved outcomes. The recurrent myocardial infarction and percutaneous coronary intervention patients were more frequent in EBT and it was beneficial for reducing CV mortality.

## 1. Introduction

Acute coronary syndrome (ACS) is the most frequent cause of the death in all cardiovascular diseases [1]. It is argued that despite readily available and highly effective treatment the mortality rates in ACS patients are still very high [2,3]. The ACS treatment guidelines have recommended that drugs for primary and secondary prevention should consist of angiotensin converting enzyme (ACE) inhibitors or angiotensin II receptors blockers (ARBs) if ACE inhibitors are not tolerated, β-blockers (BB), statins and antiaggregants (low dose aspirin or clopidogrel) [1,2,4]. The efficiency of these drugs was confirmed in large randomized clinical trials and meta-analyses. Despite available treatment and treatment recommendations ACS patients still have a high CV mortality rate, as 20% of men and 26% of women die within in 1 year [5,6]. The aim of our study is to evaluate the impact of ACS patients’ treatment, used for eight years follow-up from a first ACS episode, on cardiovascular (CV) mortality.

## 2. Materials and Methods

*Study setting and population*. A retrospective cohort study with a total of 613 patients after their first ACS episode (ST-elevated myocardial infarction (MI), non-ST-elevated MI and unstable angina) following from 2005 to 2013. Patients were involved at the Clinic of Cardiology, Hospital of Lithuanian University of Health Sciences. The ACS diagnosis was based on the ESC recommended new third universal definition of MI [7].

*Data sources and data collection.* The data received from the Hospital Medical Records Database and State Health Agencies as National Death Causes Registry and the “Sveidra” National Sickness Fund Database.

*Assessment of end-points.* By the use of unique Citizens’ ID numbers, confirmed by the birth date and gender, all the needed end-points have been collected from all databases. Patients’ death was a primary end-point. The death causes have been determined from the National Death Causes Registry and categorized by the International Statistical Classification of Diseases and Related Health Problems, Tenth revision, Australian Modification (ICD-10-AM) codes. Treatment recommendations on discharge and invasive treatments used in the first ACS episode have been collected from the Hospital Medical Records Database. Data on recurrent cardiovascular events and medical treatment used in the eight years following ACS, have been obtained from the “Sveidra” National Sickness Fund Database. Different medications have been combined by classes. An evidence-based treatment (EBT) algorithm used was created based on the guidelines from ESC experts’ opinions. The EBT covered by ACE inhibitors or ARBs, BB, statins (used in any doses) and antiaggregants. In the study, use of single medications and EBT, and the impact of this treatment on cardiovascular mortality have been evaluated. Also, the treatment and its effect on cardiovascular mortality in subgroups has been estimated. Participants who were lost during follow-up were treated as censored observations.

*Statistical analysis.* The statistical analysis has been performed by the use of the Statistical Package for Social Science (SPSS) version 13 and Microsoft Office Excel 2013 statistical programs. Descriptive statistics were used for the continuous data analysis. Categorical data are summarized as frequencies and percentages, and the chi-square test was used for the data comparison. Logistic regression and Cox regression analysis have been used for the risk assessment. First, a univariate analysis has been performed and a few the most significant survival variables (age, the history of myocardial infarction, chronic obstructive pulmonary disease, peripheral artery disease, diabetes) were included in the model. The impact of different treatments on cardiovascular mortality was analyzed using the standardized odds and hazard ratios with 95% confidence interval (CI), considering a *p* level < 0.05 as significant.

### Ethical Statement

The study was approved in 2013 by Lithuanian Bioethics Committee (No: BE-2-36) and Lithuanian National Data Protection Agency (No: 2R-279). All the patients have given their informed consent for participation in this study.

## 3. Results

The study involved 613 patients: male (395, 64.4%) and female (218, 35.6%), who experiencedtheir first ACS and were treated in 2005. The median follow-up time in this study was 7.6 years. During follow-up 48.9% of the patients (*n* = 300) died, from CV or relevant reasons (207, 69%). All baseline participant characteristics are presented in Table 1.

For monotherapy ACE inhibitors and BB, and for fixed dose combined drugs ACE inhibitors and diuretics were most frequently used. The 233 (39%) ACS survivors have been treated with PCI in the 8 year period after the first ACS episode. Treatment, according to ESC guidelines, covering combined use of ACE inhibitors or ARBs, BB, statins and clopidogrel in the 8 years follow-up from first ACS was used by 231 (37.8%) of all patients. The highest mortality rate per 100 years of observation was evaluated in patients on monotherapy, treated with ACE inhibitors (6.51/100 years of observation). In patients treated in monotherapy with new fixed dose combined ACE inhibitor and diuretic drugs, the mortality rate per 100 year of observation was higher, compared with those treated with another combined drugs (4.91/100 years of observation), and the lowest mortality rate was evaluated in patients used fixed dose ACE inhibitors and calcium (Ca) antagonists combinations.

In patients treated with PCI, the mortality rate per 100 years of observation reach 4.93. In EBT patients CV mortality was 3.8/100 years of observation. The highest impact on lowering CV mortality rate was evaluated in patients used fixed dose combination of ACE inhibitors and Ca antagonists (OR 0.045, CI 0.011–0.188, *p* < 0.001). Our study highlights that EBT use (OR 0.327, 95% CI 0.219–0.487, *p* < 0.001) in the eight year follow-up period were important in reducing CV mortality in ACS patients (Table 2).

**Table 1:** Baseline characteristics (*N* = 613).

Variables	Total *n* (%)
Age	
<70 y.o.	342 (55.7)
70–80 y.o.	209 (34.2)
>80 y.o.	62 (10.1)
Gender	
Male	395 (64.4)
Female	218 (35.6)
Medical history	
Diabetes	89 (14.5)
Hypertension	456( 74.4)
Dyslipidemia *	245 (40)
PAD	22 (3.6)
COPD	53 (8.6)
Renal insufficiency	19 (3.1)
MI history	210 (34.3)
Heart failure	233 (38)
Stroke history	40 (6.5)
Smoking	220 (35.9)
Clinical data	
Angina	450 (73.4)
PCI history	40 (6.5)
CABG history	41 (6.7)
HR > 70 b.min	343 (58)
Re-MI in 8 years	126 (21.1)
PCI treatment in 8 years	233 (39)
CV death during 8 years follow-up	300 (48.9)

y.o.—years old, PAD—periferal artery disease, COPD—chronic pulmonary disease, MI—myocardial infarction, PCI—percutaneous coronary intervention, CABG—coronary artery bypass grafting, HR—heart rate, re-MI—repeated myocardial infarction, CV—cardiovascular, b.min—beats per minute, *—total cholesterol > 5.2 mmol/L.

**Table 2:** Provided treatment during eight years and its impact on eight years cardiovascular mortality.

Treatment	Provided Treatment During 8 Years	Mortality Rate P*er* 100 Years of Observation	OR for Cardiovascular Mortality (95% CI, *p* Value)
ACE inhibitors	562 (91.7)	6.51	0.688 (0.257–1.842), 0.457
ARBs	171 (27.9)	3.85	0.474 (0.308–0.728), 0.001
BB	540 (88.1)	5.92	0.328 (0.154–0.695), 0.004
Clopidogrel	320 (52.2)	5.75	0.772 (0.526–1.133), 0.186
Statins	370 (60.4)	4.44	0.352 (0.235–0.526), <0.001
EBT	228 (37.2)	3.8	0.327 (0.219–0.487), <0.001
PCI	233 (39)	4.93	0.570 (0.381–0.853), 0.006
Ca antagonists	295 (48.1)	5.32	0.546 (0.371–0.803), 0.002
ACE inhibitors + Ca antagonists	59 (9.6)	0.44	0.045 (0.011–0.188), <0.001
ACE inhibitors + diuretics	162 (26.4)	4.91	0.668 (0.435–1.025), 0.065
ARBs + Ca antagonists	15 (2.5)	0	-
ARBs + diuretics	48 (7.8)	2.5	0.362 (0.166–0.791), 0.011
BB + diuretics	3 (0.5)	0	-
Ivabradine	18 (2.9)	0	-
Trimetazidine	122 (20)	3.79	0.514 (0.313–0.846), 0.009

- no data available. ACE inhibitors—angiotensin-converting enzyme inhibitors, ARBs—angiotensin II receptor blockers, BB—beta-blockers, Ca antagonists—calcium antagonists, EBT—use of evidence-based treatment according to the ESC guidelines, PCI—percutaneous coronary intervention, OR—odds ratio, CI—confidence interval.

In the subgroup analysis, we assessed treatment impact on CV mortality in groups of recurrent MI and PCI treatment during eight years follow-up after ACS. One hundred and twenty six (21.1%) patients experienced recurrent MI (re-MI) in the eight years following ACS, and 72 (57.1%) died. ACE inhibitors, BB, Ca antagonists, fixed dose combination of ACE inhibitors with Ca antagonists or diuretics, also ARBs with Ca antagonists or diuretics, as well as BB and diuretics, ivabradine and trimetazidine were used in the same rate in both the re-MI and non-re-MI groups, while re-MI patients were more frequently treated with ARBs (38.1% *vs.* 26.6%, *p* = 0.012), clopidogrel (81% *vs.* 47.4%, *p* < 0.001) and statins (76.2% *vs.* 59.3%, *p* = 0.001). Patients of the re-MI group had been prescribed EBT and PCI treatment (all *p* < 0.001) twice as often.

In the re-MI group only single use of fixed dose combination of ACE inhibitors and Ca antagonists, as well as statins and PCI significantly improved outcomes. For non-re-MI patients all used single drugs and PCI were associated with significantly reduced CV mortality (all *p* < 0.05). EBT recommended by guidelines used during the eight years of follow-up, was associated with significantly lower mortality rates in both groups, however this treatment used for 1 year period following ACS appeared as significant only for non-re-MI patients (OR 0.463, 95% CI 0.281–0.761, *p* = 0.002) (Table 3).

**Table 3:** Treatment for re-MI and non-re-MI groups and its’ impact on 8 year CV mortality.

Treatment	Provided Treatment During 8 Years Follow-up (*N*, %)			OR for Cardiovascular Mortality (95% CI, *p* Value)	
	re-MI Group	non-re-MI Group	*p* Value	re-MI Group	non-re-MI Group
ACE inhibitors	122 (96.8)	440 (95.2)	0.442	0.591 (0.054–6.441), 0.666	**0.328 (0.124–0.874), 0.026**
ARBs	48 (38.1)	123 (26.6)	**0.012**	0.623 (0.265–1.464), 0.277	**0.312 (0.197–0.494), <0.001**
BB	117 (92.9)	423 (91.6)	0.637	0.317 (0.036–2.821), 0.303	**0.174 (0.069–0.441), <0.001**
Clopidogrel	102 (81)	218 (47.4)	**<0.001**	0.714 (0.241–2.120), 0.544	**0.571 (0.388–0.840), 0.004**
Statins	96 (76.2)	274 (59.3)	**0.001**	**0.209 (0.062–0.707), 0.012**	**0.348 (0.233–0.521), <0.001**
EBT	77 (61.1)	151 (32.8)	**<0.001**	**0.266 (0.104–0.683), 0.006**	**0.351 (0.229–0.537), 0.001**
PCI	69 (54.8)	164 (34.7)	<0.001	0.417 (0.175–0.995), 0.049	**0.582 (0.388–0.871), 0.009**
Ca antagonists	71 (56.3)	224 (48.5)	0.118	0.445 (0.185–1.071), 0.071	**0.518 (0.352–0.762), 0.001**
ACE inhibitors + Ca antagonists	9 (7.1)	50 (10.8)	0.223	**0.058 (0.005–0.646), 0.021**	**0.056 (0.017–0.185), <0.001**
ACE inhibitors + diuretics	33 (26.2)	129 (28)	0.690	0.458 (0.105–1.987), 0.297	**0.188 (0.076–0.467), <0.001**
ARBs + Ca antagonists	5 (4)	10 (2.2)	0.255		
ARBs + diuretics	11 (8.7)	37 (8)	0.793	0.965 (0.366–2.541), 0.942	**0.504 (0.327–0.778), 0.002**
BB + diuretics	-	3 (0.6)	0.364	-	-
Ivabradine	5 (4)	13 (2.8)	0.505		
Trimetazidine	34 (27)	88 (19)	**0.051**	0.690 (0.273–1.746), 0.433	**0.429 (0.258–0.713), 0.001**

ACE inhibitors—angiotensin-converting enzyme inhibitors, ARBs—angiotensin II receptor blockers, BB—beta-blockers, Ca antagonists—calcium antagonists, EBT—use of evidence-based treatment according to the ESC guidelines, PCI—percutaneous coronary intervention, OR—odds ratio, CI—confidence interval, re-MI group—repeated myocardial infarction group, non-re-MI group—non-repeated myocardial infarction group, -—no data available. Numbers in bold—significant results.

Invasive treatment during follow-up was provided to 233 (39%) of ACS patients, and 96 (41.9%) of them died. ACE inhibitors, ARBs, Ca antagonists, fixed dose combination of ACE inhibitors with Ca antagonists or diuretics, and ARBs with Ca antagonists or diuretics, as well as BB and diuretics, ivabradine and trimetazidine were used in the same rate both in PCI and non-PCI groups. PCI group patients were prescribed more BB (*p* = 0.007), clopidogrel, statins and EBT (*p* < 0.001). In the PCI group the treatment with ARBs, statins and fixed dose combination with ACE inhibitors and Ca antagonists or ARBs and diuretics was associated with a significantly lower rate of CV mortality. For non-PCI patients who used single ARBs, BB, Ca antagonists, trimetazidine, and also fixed dose combinations of ACE inhibitors with Ca antagonists and diuretics, and ARBs and diuretics had a significant impact on CV mortality rate. The use of EBT during the eight years follow-up period was associated with strong CV mortality reduction in both groups with stronger evidence for PCI group (OR 0.307, 95% CI 0.172–0.548, *p* < 0.001) (Table 4).

A Cox proportional hazards model, standardized by a few significant factors was used to evaluate the use of ARBs, BB, statins, calcium antagonists, trimetazidine and fixed dose combined treatment with ACE inhibitors and calcium antagonists and ARBs and diuretics as significant for CV mortality, all *p* < 0.05. The use of EBT in the eight years following ACS was associated with significant decrease in CV mortality (HR 0.541, CI 0.394–0.742, *p* < 0.001). For the patients in the re-MI group according to the Cox model only the use of BB and EBT were significantly important in decreasing CV mortality. In non-re-MI patients, contrary to the logistic model ACE inhibitors and clopidogrel were not significant for CV mortality rate. In the PCI group Cox regression analysis showed a significant ACE inhibitors, BB, ARBs, statins, trimetazidine and fixed dose combination of ACE inhibitors and calcium antagonists effect on CV mortality (all *p* < 0.05). For the non-PCI group the results were the same as those of the logistic regression, except for fixed dose combined treatment with ACE inhibitors and calcium antagonists. Trimetazidine also appeared as not significant (Table 5).

**Table 4:** Treatment in PCI and non-PCI groups and its impact on eight year cardiovascular mortality.

Treatment	Provided Treatment During 8 Years (*N*, %)		*p* Value	OR for Cardiovascular Mortality (95% CI, *p* Value)	
	PCI-Group	non-PCI-Group		PCI-Group	non-PCI-Group
ACE inhibitors	220 (96.1)	342 (95.3)	0.643	0.384 (0.092–1.610), 0.191	0.321 (0.096–1.072), 0.065
ARBs	77 (33.6)	94 (26.2)	0.053	0.407 (0.222–0.746), 0.004	0.404 (0.241–0.675), 0.001
BB	219 (95.6)	321 (89.4)	0.007	0.345 (0.083–1.445), 0.145	0.151 (0.049–0.468), 0.001
Clopidogrel	183 (80.3)	137 (38.3)	<0.001	0.560 (0.283–1.108), 0.096	0.908 (0.570–1.446), 0.683
Statins	172 (75.1)	198 (55.2)	<0.001	0.282 (0.147–0.542), <0.001	0.464 (0.291–0.739), 0.001
EBT	143 (62.7)	85 (23.7)	<0.001	0.307 (0.172–0.548), <0.001	0.547 (0.322–0.926), 0.025
Ca antagonists	118 (51.5)	177 (49.3)	0.599	0.682 (0.394–1.180), 0.171	0.452 (0.285–0.718), 0.001
ACE inhibitors + Ca antagonists	23 (10)	36 (10)	0.995	0.093 (0.021–0.419), 0.002	0.044 (0.010–0.188), <0.001
ACE inhibitors + diuretics	61 (26.8)	101 (28.1)	0.716	0.603 (0.322–1.131), 0.115	0.568 (0.345–0.935), 0.026
ARBs + Ca antagonists	6 (2.6)	9 (2.5)	0.932		
ARBs + diuretics	19 (8.3)	29 (8.1)	0.925	0.247 (0.069–0.887), 0.032	0.249 (0.102–0.607), 0.002
BB + diuretics	3 (1.3)		0.030		
Ivabradine	13 (5.7)	5 (1.4)	0.003		
Trimetazidine	66 (28.8)	56 (15.6)	<0.001	0.599 (0.322–1.114), 0.105	0.477 (0.257–0.884), 0.019

ACE inhibitors—angiotensin-converting enzyme inhibitors, ARBs—angiotensin II receptor blockers, BB—beta-blockers, Ca antagonists—calcium antagonists, EBT—use of evidence-based treatment according to the ESC guidelines, PCI—percutaneous coronary intervention, OR—odds ratio, CI—confidence interval, PCI group—percutaneous coronary intervention group, non-PCI group—non-percutaneous coronary intervention group.

**Table 5:** Treatment impact on CV mortality by Cox proportional hazards model.

Treatment	HR for CV Mortality, (95% CI, *p* Value)	HR for CV Mortality, (95% CI, *p* Value) re-MI Group	HR for CV Mortality, (95% CI, *p* Value) non-re-MI Group	HR for CV Mortality, (95% CI, *p* Value) PCI Group	HR for CV Mortality, (95% CI, *p* Value) non-PCI Group
ACE inhibitors	0.798 (0.384–1.660), 0.546	0.655 (0.080–5.340), 0.693	0.479 (0.214–1.075), 0.074	**0.218 (0.061–0.781), 0.019**	0.858 (0.331–2.224), 0.752
ARBs	**0.479 (0.343–0.669), <0.001**	0.602 (0.334–1.086), 0.092	**0.367 (0.238–0.565), <0.001**	**0.546 (0.311–0.958), 0.035**	**0.399 (0.261–0.610), <0.001**
BB	**0.567 (0.363–0.884), 0.012**	**0.117 (0.040–0.342), <0.001**	**0.509 (0.296–0.878), 0.015**	**0.248 (0.097–0.638), 0.004**	**0.530 (0.305–0.919), 0.024**
Clopidogrel	0.860 (0.650–1.138), 0.291	0.618 (0.301–1.268), 0.189	0.726 (0.522–1.011), 0.058	0.765 (0.407–1.438), 0.406	0.890 (0.629–1.261), 0.513
Statins	**0.495 (0.373–0.655), <0.001**	0.694 (0.362–1.332), 0.272	**0.387 (0.278–0.539), <0.001**	**0.344 (0.204–0.581), <0.001**	**0.521 (0.370–0.734), <0.001**
EBT	**0.541 (0.394–0.742), <0.001**	**0.454 (0.253–0.813), 0.008**	**0.447 (0.300–0.667), <0.001**	**0.414 (0.242–0.707), 0.001**	**0.556 (0.360–0.860), 0.008**
Ca antagonists	**0.666 (0.503–0.881), 0.004**	0.637 (0.344–1.181), 0.152	**0.559 (0.400–0.782), 0.001**	0.711 (0.428–1.180), 0.187	**0.577 (0.409–0.814), 0.002**
ACE inhibitors + Ca antagonists	**0.071 (0.018–0.285), <0.001**	0.175 (0.024–1.299), 0.088	**0.042 (0.006–0.303), 0.002**	**0.179 (0.043–0.741), 0.018**	-
ACE inhibitors + diuretics	**0.667 (0.477–0.932), 0.018**	0.975 (0.494–1.925), 0.942	**0.563 (0.377–0.839), 0.005**	0.619 (0.328–1.168), 0.139	**0.624 (0.417–0.935), 0.022**
ARBs + Ca antagonists	-	-	-	-	-
ARBs + diuretics	**0.417 (0.213–0.819), 0.011**	0.548 (0.192–1.567), 0.262	**0.307 (0.125–0.754), 0.010**	0.479 (0.148–1.554), 0.221	**0.351 (0.153–0.805), 0.013**
BB + diuretics	-	1.362 (0.714–2.595), 0.348	-	-	**1.504 (1.000–2.262), 0.050**
Ivabradine	-	-	-	-	-
Trimetazidine	**0.598 (0.403–0.889), 0.011**	0.948 (0.511–1.759), 0.866	**0.383 (0.220–0.667), 0.001**	**0.501 (0.260–0.966), 0.039**	0.633 (0.384–1.044), 0.073

ACE inhibitors—angiotensin-converting enzyme inhibitors, ARBs—angiotensin II receptor blockers, BB—beta-blockers, Ca antagonists—calcium antagonists, EBT—use of evidence-based treatment according to the ESC guidelines, HR—hazard ratio, CI—confidence interval, re-MI group—repeated myocardial infarction group, non-re-MI group—non-repeated myocardial infarction group, PCI group—percutaneous coronary intervention group, non-PCI group—non-percutaneous coronary intervention group, -—no data available. Numbers in bold—significant results.

## 4. Discussion

*Principal results.* 126 patients experienced recurrent MI in the eight years follow-up, invasive treatment was provided to 233 (39%) and 48.9% of the patients (*n* = 300) died, 207 (69%) of them from cardiovascular or relevant reasons. Treatment according to ESC guidelines in the eight years of follow-up from first ACS was used by 231 (37.8%) of all patients. Single use of ARBs, BB, statins, Ca antagonists, fixed dose combination of ACE inhibitors and Ca antagonists, and also ARBs and diuretics, and trimetazidine significantly reduce CV mortality in ACS patients during the eight years of follow-up. The study highlighted, that treatment with ACE inhibitors or Ca antagonists, ARBs, BB, and clopidogrel as single drug treatment was not significant for CV mortality in the re-MI group, however the combined use of these drugs significantly improved treatment outcomes. Our study highlights EBT use (OR 0.327, 95% CI 0.219–0.487, *p* < 0.001 or HR 0.541, CI 0.394–0.742, *p* < 0.001) and PCI treatment (OR 0.570, 95% CI 0.381–0.853, *p* = 0.006) in the eight years follow-up period were important in reducing CV mortality in ACS patients.

*Interpretation of study results.* According to available evidence, based on large clinical trials and meta-analysis, the EBT is beneficial for all patients following ACS. First of all our study highlighted insufficient use of EBT during follow-up. This is similar to some other studies which showed the EBT prescription level was only up to 58% in different countries [8,9,10,11]. There are a lot of reasons for non-compliance, such as non-prescription, complications associated with drug use, out-of-pocket treatment costs and others [10,12]. Secondly, in accordance to previous clinical trials, our study confirmed the benefits of combined drug therapy when used in ACS patients [2,10,12,13,14,15,16,17].

Thirdly, this study highlighted the effects of the provided treatment on CV mortality in different subgroups of patients who experienced recurrent MI or PCI in the eight year period after ACS. We found that some drugs used in re-MI patients were more frequently prescribed, such as ARBs and clopidogrel, while these drugs were not associated with improved CV mortality. In subgroup analysis fixed dose drugs combinations and combined use of EBT was also more beneficial than the same drugs used in monotherapy. The increased use of clopidogrel in re-MI patients is explainable by the dual antiaggregants therapy, proved to be beneficial following acute phase of MI, while increased use of ARBs can be explained by the novelty of these drugs in Lithuania in this period. Our study is in agreement to some other studies and confirms that re-MI and PCI patients were more frequently on EBT and it was beneficial for reducing CV mortality. Risk reduction in CV mortality can be up to 67%–75% [18,19,20].

*Strengths and limitations*. The most important limitation of this study is its retrospective design which limits studying the reasons of treatment failure. Some previous studies have described possible associations to treatment failure, such as common comorbidities (asthma and rhythm and conduction disorders for BB use), occurring side-effects (allergy), and contraindications (renal failure for ACE inhibitors or ARB use), however patients and physicians non-compliance could be significant factors as well.

From other side, strength of our study was the possibility to trace all required end-points of each patient in different available databases using the unique Citizen’s ID Number. The identity of each patient was confirmed using gender and birth date. This allowed us to use data mining tools and get most reliable data for the primary and secondary end-points in this study.

*Possible implications to future practice.* The study highlighted the high rate of CV mortality in ACS patients and clear evidence of the benefits from EBT use. The use of combined treatments has large benefits for recurrent MI patients and patients who have experienced PCI, so for these subgroups should be used more widely. Low rate of compliance to EBT should be further investigated and has to be improved in future practice.

## 5. Conclusions

In an eight year follow-up 48.9% of the study participants died, and 69% of these died from cardiovascular or related reasons. Some drugs used in monotherapy were not associated with lower CV mortality rates. The combined use of drugs, according to ESC guidelines, significantly improved treatment outcomes. The recurrent MI and PCI patients were more frequently treated according to ESC guidelines and this action was shown to be beneficial in reducing CV mortality.
